# Aberrant patterns of brain cerebral blood flow in Chinese han first-episode drug-naïve depressive patients with and without a family history of depression

**DOI:** 10.18632/oncotarget.20306

**Published:** 2017-08-17

**Authors:** Shikai Wang, Lina Wang, Ping Jing, Ping Guo, Weifang Zheng, Jie Li, Mincai Qian

**Affiliations:** ^1^ Department of Psychological Medicine, Huzhou Third People’s Hospital, Huzhou, China; ^2^ Department of Psychological Medicine, Tianjin Anding Hospital, Tianjin, China; ^3^ Department of Psychological Medicine, Wenzhou Seventh People’s Hospital, Wenzhou, China

**Keywords:** depression, family history, pCASL, regional cerebral blood flow, aberrant pattern

## Abstract

A positive family history plays a key role in the brain pathology of depression patients and previous research has confirmed that disturbed mood maintenance may be related to abnormal regional cerebral blood flow (rCBF). However, little is known about whether the rCBF is different between depression patients with and without family histories. To address this question, we examined the rCBF in drug-naïve, first-episode depression patients with and without family histories of depression using a 3D pseudo-continuous arterial spin-labelling technique. We found that decreased rCBF was predominantly observed in the patients without family histories, while decreased and increased rCBF co-existed in patients with family histories. The observed brain regions with altered rCBF were associated with affection processing, such as the prefrontal, occipital and insular areas. However the patterns of rCBF alteration observed in the present study were different from those found in previous studies where patients were compared with healthy controls. Our present findings, together with the findings from previous studies have prompted the need of a long-term follow-up study to characterize the brain features of the developmental trajectory of depression and investigate the targets for precise, personalized treatments.

## INTRODUCTION

Many previous studies confirmed that genetics play a key role in the etiology of major depressive disorder [1, 2] and many previous studies confirmed that depression patients with a positive family history are more inclined to develop treatment refractory depression [3]. Depression in patients with a positive family history is usually considered to be mainly associated with genetic factors, but depression in patients with a negative family history is usually attributed to environmental factors [4]. More notably, some studies have reported that differences can be observed in the expression of depressive symptoms, cognitive impairments, and treatment outcomes between depression patients with and without family histories [5, 6].

Several previous neuroimaging studies based on Magnetic Resonance Imaging (MRI) have focused on structural and functional alterations between depression patients and healthy controls. For example, one previous study found that some brain regions located in the prefrontal area and the limbic system demonstrated structural aberrations and that the connectivity between these brain regions was also affected, including the ventromedial prefrontal cortex, the dorsolateral prefrontal cortex, the anterior cingulate cortex, the lateral orbital prefrontal cortex, the amygdala, the hippocampus and the ventral striatum [7]. At the same time, some studies reported that the functional connectivity between the amygdala and the anterior cingulate cortex regions is also unusual in depression patients compared to healthy controls [8]. Furthermore, a previous study found that the regional blood flow abnormally increased in the ventromedial prefrontal cortex and the lateral orbital prefrontal cortex, but decreased in the dorsolateral prefrontal cortex in depression patients [9]. Additionally, many studies have been confirmed that local spontaneous neural activity is abnormal in depression patients, including amplitude of low frequency fluctuation (ALFF), regional homogeneity (Reho), functional connectivity density (FCD), etc. [10–14]. For example, previous study found that decreased ReHo were located in the left insula, superior temporal gyrus, inferior frontal gyrus, lingual gyrus and cerebellumanterior lobe in the patients with treatment resistant depression (TRD), simultaneously, increased ReHo were found in the left superior temporal gyrus, cerebellum posterior lobe, cerebellum anterior lobe, the right cerebellar tonsil and bilateral fusiform gyrus in the TRD patients [15]. This study supported the hypothesis that the abnormality hypothesis of limbic-cortical networks in major depressive disorder (MDD) [16]. Liu et al. found that patients with MDD exhibited significantly decreased fractional amplitude of low frequency fluctuation (fALFF) in right cerebellum posterior lobe, left parahippocampal gyrus and right middle frontal gyrus and increased fALFF in left superior occipital gyrus/cuneus [17]. Guo et al. found that exhibited reduced fractional amplitude of low-frequency fluctuations in the right middle frontal gyrus (orbital part) and decreased network homogeneity in the left middle frontal gyrus [18, 19]. Zhuo et. found that decreased global FCD was located in the left postcentral and precentral gyri, right fusiform gyrus and lingual gyrus [20]. Taken together, these findings inclined to support the hypothesis that decreased regional activity and network homogeneity in the frontal cortex may be the key impairment of the fronto-limbic network in the patients with MDD [19]. Collectively, all of the abovementioned findings converge to suggest that the brain regions that participate in emotion regulation, reward regulation, memory and executive function regulation all show structural and functional abnormalities and cerebral blood follow disturbances.

Certain studies have found that cerebral perfusion alterations are predominantly located in the anterior cingulate cortex, right medial prefrontal cortex, temporal cortex, hippocampus, thalamus and cerebellum [21, 22]. Vasic found that CBF was reduced in the anterior cingulate and bilateral parahippocampal areas and increased in the frontoparietal and striatal regions in MDD patients [23]. Drevets summarized that the brain rCBF in patients with depression showed a general tendency to decrease and that the brain regions with abnormal CBF in depression patients predominantly included the frontal lobe, temporal lobe, cingulate cortex, amygdala, caudate nucleus, and paralimbic system [14]. All the aforementioned studies converged to suggest that the alterations of CBF in the regions located in the limbic-subcortical-cortical circuit appear to be common brain characteristics of patients with depression [24]. However, although previous findings related to rCBF enhanced our understanding of the pathological features of major depressive disorder, few studies have reported the difference between depression patients with and without family histories.

The pCASL technique is a recently developed method used to acquire information on cerebral blood flow. In the last five years, many studies adopted pCASL to study mental disorders and discovered many important findings [22, 25]. Based on previous studies, in the current study, we tried to investigate the cerebral blood flow differences in drug-naïve, first-episode depression patients with and without family histories using the 3D pCASL technique [26]. We hypothesized that the aberrant pattern of rCBF is different between these two groups, the rCBF alterations in the MDD patients with a family history will more widely and complexity than the MDD patients without a family history.

## RESULTS

### Comparison of the brain regions with altered cerebral perfusion between depression patients with a family history of depression and the healthy control group

**Figure 1:**
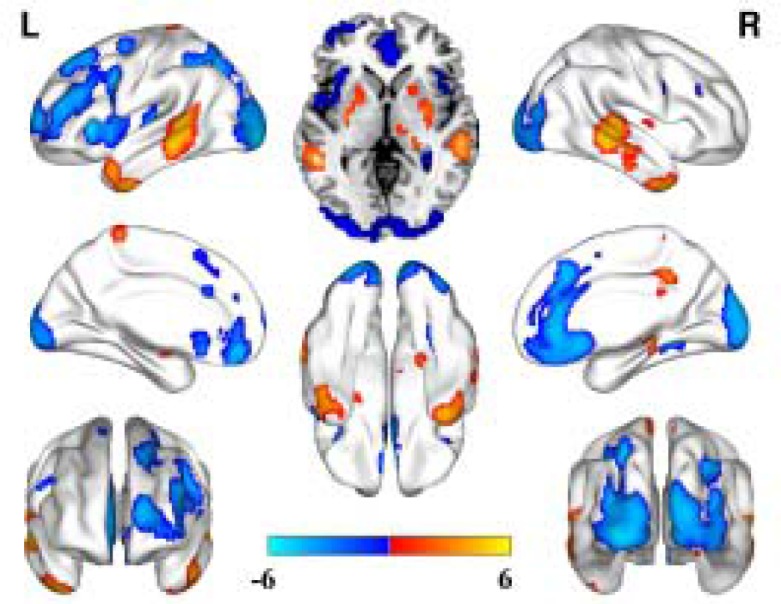
CBF alterations in the depression patients with a family history compared to the healthy controls (FDR correction, *P* < 0.05, cluster size ≥ 50)

Compared to the healthy controls, depression patients with a family history showed significantly increased CBF in the bilateral basal ganglia, the bilateral middle temporal gyrus, the lateral inferior gyrus, the left paracentral gyrus, and the right thalamus. Significantly decreased CBF was observed in the bilateral prefrontal lobe, the insular, the occipital lobe and the right medial temporal lobe (Figure 1).

### Comparison of the brain regions with altered cerebral perfusion between depression patients without a family history of depression and the healthy control group

**Figure 2:**
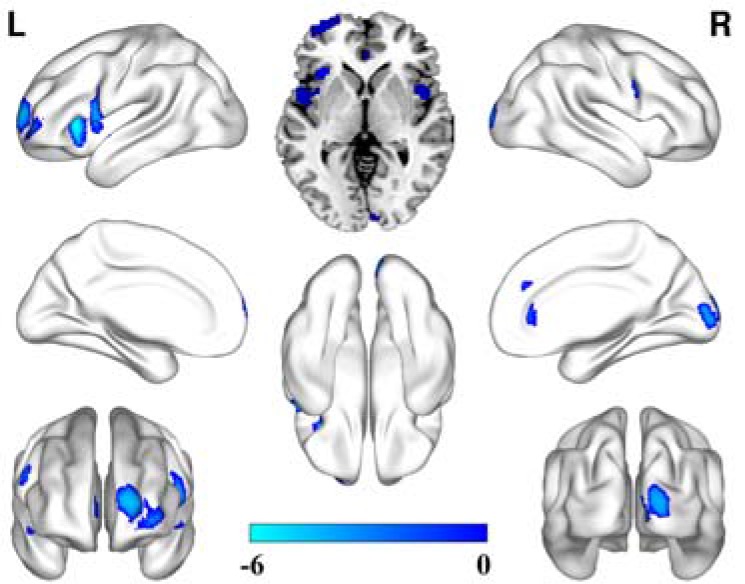
CBF alterations in the depression patients without a family history compared to the healthy controls (FDR correction, *P* < 0.05, cluster size ≥ 50)

Compared to the healthy controls, unlike the depression patients with a family history, significantly decreased rCBF was only observed in the bilateral insular, the left prefrontal lobe and the right occipital lobe. No significantly increased CBF brain regions were observed in these patients without a family history (Figure 2).

### Relationship between rcbf alterations and hamd scores

We calculated the correlations between the rCBF alterations and HAMD scores in the patients with and without family histories. Unfortunately, we did not find any correlations.

## DISCUSSION

In current study, we first adopted pCASL methods to explore rCBF abnormalities in first-episode, drug-naïve depression patients with and without family histories of depression. We found distinct rCBF alterations between the depression patients with and without family histories compared to the healthy controls. The most important finding in our present study is that we found decreased rCBF only in the depression patients without a family history compared to the healthy controls. However, decreased and increased rCBF co-existed in the patients with a family history. The distribution of rCBF alterations in the depression patients with a family history is more widespread than in the patients without a family history. In addition, increased rCBF is also evident in the patients with a family history. In summary, our findings suggest that the depression patients with a family history had more extensive cerebral blood disturbance than the patients without a family history.

A strength of our study is that we enrolled drug-naïve, first-episode patients. Therefore, our findings were unlikely to be influenced by confounding factors (such as previous therapy). This allows our findings to more objectively reflect the pathological features of these patients. The common rCBF alteration in the two patient groups was the decreased rCBF in the prefrontal lobe, the insular and the occipital lobe areas. The specific alteration in the patients with a family history was the increased rCBF in the bilateral basal ganglia, the bilateral middle temporal gyrus, the lateral inferior gyrus, the left paracentral gyrus, the right thalamus, these findings supported the hypothesis that increased cerebellar-default-mode-network connectivity in MDD patients to some extent [27]. Previous studies confirmed that functional or structural alterations in the prefrontal-limbic system participated in the regulation of affection, the regulation of memory processes and executive function [7, 28–31]. Previous studies have also reported that decreased rCBF was also found in the prefrontal cortex and the orbital prefrontal cortex and that these brain regions participated in the regulation of aggression, sexual functioning and perseverative affection processing [23, 31–33]. In addition, decreased rCBF in the insular in our present study supported the hypothesis that insular act as an integration center of emotional processing which is disrupted in the depressed patients [34]. Most of our current findings are consistent with the previous studies and together support the hypo-metabolism hypothesis of depression.

However, some inconsistencies were also observed between our current study and previous studies. For example, we found increased rCBF in the bilateral basal ganglia, the bilateral middle temporal gyrus, the lateral inferior gyrus, the left paracentral gyrus and the right thalamus. This finding did not completely support the previous hypothesis to some extent [7, 27–34]. However, this is not entirely incomprehensible. About the rCBF alterations in MDD patients, inconsistent findings reported by different studies, to the best of our knowledge, there is none a completely consistent findings was reported by two studies. Just as Drevets summarized and reported by the meta-analysis of Gong, the brain rCBF in MDD patients demonstrated a general decrease tendency which predominantly located in the limbic-subcortical-cortical circuit [14, 24]. Simultaneously, in these studies included in the meta-analysis and review, also some studies reported increased rCBF in some brain regions and many inconsistent findings among these studies [14, 27]. We cannot completely explain this difference based on our current knowledge. The probably reasons we can suggest are caused by many influence factors’ differences in different studies, such as patient differences, MRI parameters, antidepressants, et al. [35, 36]. The inconsistent findings provide pivotal information for us to consider that we should conduct a long-term follow-up study which adopt a uniform enrolled criteria and uniform MRI parameters to test this and to explore the dynamic alterations in drug naive depression patients from the first episode to acquired appropriate treatments. Then, we can characterize the brain features of the developmental trajectory of depression and investigate the treatment targets for precise, personalized treatments.

A unique phenomenon was observed in our study. When we compared the two patient groups, we found that patients with a family history exhibited increased rCBF in the bilateral middle temporal and the hippocampus brain regions, but this difference cannot withstand the FDR correction. This observation also warrants further investigation. The reasons for the lack of differences between the patients with and without a family histories in the current study can be explained as follows. First, genetic influences on rCBF alterations have rarely been reported in previous studies, warranting further investigations. Second, as previous studies reported that rCBF was influenced by many factors such as age, gender, illness duration, MRI parameters, et al. [35], this may account for our current findings. Third, our present study had a flawed design. We need a well-designed study to further explore the differences between these groups so that we can provide more useful information to understand the pathological mechanisms of MDD patients with and without family histories and propose potential mechanisms underlying the influence of genetic factors on rCBF.

### Limitation

Cardiac noise, respiratory noise, low frequency fluctuations and artefacts from physiological noise are especially prevalent in the frequency bands examined at rest. Therefore, Murphy K et al. advised a pre-processing step to remove physiological noise from the data using simultaneously collected pulse and respiration data [35]. From this perspective, we must collect physiological data in the study. This is a limitation of our present study, and we must consider these factors in the future study. More interestingly, previous studies found that pulse and respiration can influence the CBF measurement in some special conditions. For example, using volunteers, Kolbitsch reported that continuous positive airway pressure (CPAP) (12 cm H2O) can increase cerebral perfusion [36]. However, Zelaya confirmed that no correlation was found between regional CBF changes and respiratory rate or heart rate [37]. Therefore, the effects of pulse and respiration on CBF are inconsistent in different studies, thus warranting further study for clarification. In the present study, we did not collect pulse and respiration data in the MDD patients and healthy controls. This decision may represent a limitation to some extent, but these factors cannot completely elicit the physiological noise influence. We must conduct future research according to Murphy K’s suggestion. Eklund et al. proposed that a voxel defining threshold of 0.001 is probably a reasonable control under current RFT options, though non-parametric tests would be preferable [38]. In current study, when we adopted this method, all the significant differences were disappeared, this is of great concern, in the future study, we must adopt the Eklund et al. proposed methods to analysis the data to acquire more accurately findings.

## MATERIALS AND METHODS

### Subjects

All of the patients were diagnosed by two senior professional psychiatrists who adopted the SCI-D according to the criteria for major depressive disorder at the first episode in the DSM-IV (TR Version). An additional criterion was that the patients had not accepted any regular therapeutic or physical treatments in the last two weeks. Another additional criterion was that the family history was also diagnosed by two senior professional psychiatrists according to guardian’s reports and medical records. All of the healthy controls were excluded by two professional psychiatrists who adopted the SCI-D (NP Version). The 17-item version Hamilton Rating Scale for Depression (HAMD) [39] was used to assess the severity of the depressive symptoms in the patients. A total of 83 patients were enrolled from January 2015 to December 2016 in the current study. The mean age of the patients with a positive family was 33.6 ± 7.9 years and the mean illness duration was 130.8 ± 93.8 (days). The mean age of the patients with a negative family history was 34.7 ± 6.5 years and the mean illness duration was 116.2 ± 95.4 (days). A total of 47 healthy controls were recruited from the hospital staff of Huzhou 3rd Hospital (Huzhou, Zhejiang) and Wenzhou seventh people’s hospital (Wenzhou, Zhejiang), with a mean age of 33.0 ± 7.7 years. Two senior psychiatrists ruled out a diagnosis of potential mental disorder and the volunteers were only enrolled if they did not have a positive family history of mental disorder. No significant differences were observed among the three groups with regard to age or gender (Table 1). The common exclusion criteria were as follows: participants with a history of unconsciousness for ≥ 5 min; a neurological disease diagnosis history; a severe mental disorder such as schizophrenia; drug abuse; serious physical illness; patients who were pregnant or lactating; participation in any other research study; treatment within the last two weeks; or endocrine disease and other contraindication for MRI scanning. The Ethics Committee of Hu Zhou 3rd Hospital and Wenzhou Seventh people’s Hospital all approved the current study. All participants fully understood the current study purposes and provided written informed consent.

**Table 1:** Demographic and clinical characteristics of the participants

	FDD+PF (*n* = 47)	FDD+NF (*n* = 36)	HC (*n* = 47)	*P*-value
Age (yrs)	33.6 ± 7.9	34.7 ± 6.5	33.0 ± 7.7	0.600
Sex (M/F)	28/19	20/16	27/20	0.934
Illness duration, month	130.8 ± 93.8 (days)	116.2 ± 95.4 (days)		0.488
HRSD score	37.21 ± 11.24	27.78 ± 7.64		< 0.001

FDD+PF: First-episode, drug-naïve depression patients with a positive family history; FDD+NF: First-episode, drug-naïve depression patients with a negative family history; HC: healthy controls; HRSD: 17-item version Hamilton Rating Scale for Depression.

### Methods

#### MRI image

We used the Philips Achieva 3.0 T MRI system (Philips Medical Systems Nederland B.V., The Netherlands) to perform the scans. The participants were arranged in a comfortable position and were given special earplugs to minimize the impact of noise during the scanning. A foam pad was placed around each participant’s head to control head movement. The scope of MRI scanning included the whole brain. The pCASL scanning parameters were as follows: Echo time/repetition time, 4,000 ms/14 ms; slice thickness, 7 mm; slice number, 17; matrix = 80 × 80; interlayer spacing, 1 mm; voxel size, 3 × 3 × 7 mm; label spacing, 20 mm; labeling duration, 1,650 ms; label delay, 1,525 ms; and FOV (field of view), 240 × 240 mm.

### Functional MRI data preprocessing

In the present study, common data processing software for brain functions were used, including Statistics Parameter Mapping (SPM8; Wellcome Department of Imaging Neuroscience, London, UK), and the Relative Expression Software Tool was used to conduct the data preprocessing. The specific process consisted of the following steps. First, for data format conversion, the ASL images from the scans were processed with the built-in software for digital subtraction to obtain the perfusion map and the format was then converted to a dual file format for SPM processing. This step was completed with MRICroN software 1.40 (http://www.cabiatl. com/mricro/mricro/mricro.html). Second, for spatial normalization, the individual perfusion image was input into a standard PET template provided by SPM8. Third, for the removal of non-brain tissues, the normalized image was computed with a standard mask to remove non-brain tissue. This process was performed using the ImCalc function of SPM8. Fourth, for Gaussian smoothing, a Gaussian kernel of 4×4×4 mm3 full width at half maximum (FWHM) was used to perform the spatial smoothing of the images, reduce the remaining inter-individual differences after normalization, and improve the SNR. Fifth, mean whole brain perfusion was extracted to obtain a normalized image for each individual. The inter-individual differences in brain perfusion were removed using the individual whole brain perfusion information/average individual whole brain perfusion information. This was performed by the REST and the ImCalc functions of SPM8. An index of transient head motion (AFNI’s@1dDiffMag) was calculated from each subject’s motion parameters for use as a nuisance covariate in the group-level analyses.This was performed by the REST and the ImCalc functions of SPM8.

### Statistical analyses

SPM8 was used to conduct the statistical analyses of the brain perfusion data between the two groups and to demonstrate the final results. An independent one-sample *t*-test was conducted for the healthy control and patient groups. Age and gender were considered as the covariates to generate the difference map of cerebral blood perfusion between any two groups. The results were put into a single T1 template using the xjView software. Difference comparisons were corrected by the false discovery rate (FDR) method with a significance threshold of *P* < 0.05.

SPSS 19.0 statistical analysis software (SPSS, Inc., Chicago, IL, USA) was adopted for all statistical analyses of all data from the two groups. The statistical analyses were performed using two independent sample *t*-tests and *P* < 0.05 was considered to indicate a statistically significant difference. The data are presented as the mean ± standard deviation.

## CONCLUSIONS

In conclusion, we found that compared to healthy controls, the rCBF alteration patterns in drug-naïve first-episode depression patients with and without family histories were different. Decreased cerebral perfusion was predominantly observed in the patients without family histories, but decreased and increased rCBF co-existed in the patients with family histories. Compared to healthy controls, the MDD patients with and without family histories all showed decreased rCBF in the prefrontal lobe, the insular, and the occipital lobe. These altered rCBF brain regions were related to the brain regions that are associated with affection processing. The more important finding in our study is that there are no differences between MDD patients with and without family histories of depression after FDR correction. These critical counterintuitive findings in the present study have prompted us to conduct a long-term follow-up study and avoid confounding factors in first-episode, drug-naive depression patients to acquire appropriate treatments. Therefore, we can characterize the brain features of the developmental trajectory of depression and investigate the treatment targets for precise, personalized treatments.
